# Identification of key genes and pathways in adrenocortical carcinoma: evidence from bioinformatic analysis

**DOI:** 10.3389/fendo.2023.1250033

**Published:** 2023-11-20

**Authors:** Mengsha Yin, Yao Wang, Xinhua Ren, Mingyue Han, Shanshan Li, Ruishuang Liang, Guixia Wang, Xiaokun Gang

**Affiliations:** ^1^ Department of Endocrinology and Metabolism, The First Hospital of Jilin University, Changchun, China; ^2^ Department of Orthopedics, The Second Hospital Jilin University, Changchun, China

**Keywords:** adrenocortical carcinoma, gene expression omnibus, differentially expressed genes, protein-protein interaction, Kaplan-Meier curve

## Abstract

Adrenocortical carcinoma (ACC) is a rare endocrine malignancy with poor prognosis. The disease originates from the cortex of adrenal gland and lacks effective treatment. Efforts have been made to elucidate the pathogenesis of ACC, but the molecular mechanisms remain elusive. To identify key genes and pathways in ACC, the expression profiles of GSE12368, GSE90713 and GSE143383 were downloaded from the Gene Expression Omnibus (GEO) database. After screening differentially expressed genes (DEGs) in each microarray dataset on the basis of cut-off, we identified 206 DEGs, consisting of 72 up-regulated and 134 down-regulated genes in three datasets. Function enrichment analyses of DEGs were performed by DAVID online database and the results revealed that the DEGs were mainly enriched in cell cycle, cell cycle process, mitotic cell cycle, response to oxygen-containing compound, progesterone-mediated oocyte maturation, p53 signaling pathway. The STRING database was used to construct the protein–protein interaction (PPI) network, and modules analysis was performed using Cytoscape. Finally, we filtered out eight hub genes, including CDK1, CCNA2, CCNB1, TOP2A, MAD2L1, BIRC5, BUB1 and AURKA. Biological process analysis showed that these hub genes were significantly enriched in nuclear division, mitosis, M phase of mitotic cell cycle and cell cycle process. Violin plot, Kaplan-Meier curve and stage plot of these hub genes confirmed the reliability of the results. In conclusion, the results in this study provided reliable key genes and pathways for ACC, which will be useful for ACC mechanisms, diagnosis and candidate targeted treatment.

## Introduction

Adrenocortical carcinoma (ACC) is a rare endocrine malignancy with poor prognosis (1). According to the new WHO criteria, ACC is subclassified according to its morphological characteristics, including conventional, oncocytic, myxoid and sarcomatoid subtypes (2).The incidence of ACC is estimated to be 1 to 2 per million per year (3). Women appear to have a slightly higher incidence rate compared to men, with a ratio of 1.5-2.5 to 1 (1). ACC mainly presents as an incidental imaging finding (4), and unfortunately, the 5-year survival rate is less than 40% in most literature reports (5–8). However, there are few treatments available for this disease with such low survival rates (9). At present, surgical resection is considered the primary approach for treating ACC. Complete removal of the primary tumor is crucial for improving prognosis, while 5-year survival rates are as low as 20% and 10%, respectively, for patients with microscopic or macroscopic involvement at the tumor margin (6). However, despite undergoing complete resection, many patients with ACC experience recurrence and disease progression, so adjuvant therapy is often recommended. The medication that is commonly used for adjuvant therapy is mitotane, which is the only approved treatment for advanced ACC, but it is less effective in the advanced stages of the disease and has toxicity and side effects (10). Another effective adjuvant therapy is radiotherapy, which has been shown to reduce local recurrence rates but not improve clinical outcomes (11–14). Overall, regardless of the treatment, the prognosis for advanced ACC remains poor. In recent years, there have been ongoing efforts to gain a deeper understanding of ACC in terms of its tumorigenesis and progression mechanisms. For example, a genome-wide methylation analysis shed light on the prognostic implications of hypermethylated ACC. It was found that hypermethylation in ACC primarily leads to the silencing of specific tumor suppressor genes, thereby contributing to a poorer prognosis (15). This research highlights the importance of epigenetic alterations, specifically DNA methylation, in ACC. Transcriptomic analysis has revealed that somatic inactivation mutations of the tumor suppressor gene TP53 and activation mutations of the proto-oncogene β-catenin (CTNNB1) are the most frequently observed mutations in ACC, these mutations have been associated with a poor prognosis in ACC patients (16). Whole-exome sequencing has also been utilized to genetically characterize potential somatic mutations in ACC, in order to obtain genomic landscape of ACC and facilitate the identification of frequently altered pathways, which may guide targeted therapy research (17). However, it is concerning that the various advancements in the international scientific community in the field of epigenetics, transcriptomics, and exome sequencing have not been reported. Consequently, there is an urgent need to identify new candidate targets for ACC. To address this gap, it is crucial to explore the potential of high-throughput techniques such as RNA-sequencing and microarrays, which have enabled significant insights into the molecular mechanisms of tumors over the past few decades. By analyzing DEGs and functional pathways between tumor and normal tissues, we can gain a deeper understanding of the pathogenesis and progression of tumor. In this study, we conducted an integrated bioinformatics analysis to identify hub genes and pathways in ACC, which can greatly contribute to our understanding of the underlying mechanisms and candidate targeted therapy options for ACC.

## Materials and methods

### Microarray data

Gene expression profiles of ACC were obtained from the GEO database (https://www.ncbi.nlm.nih.gov/geo/). The screening criteria were as follows: i) adrenocortical carcinoma, ii) samples containing tumor and normal adrenal gland tissues, iii) expression profiling by array and iiii) Homo sapiens. A total of three GEO datasets were selected, including GSE12368 (18), GSE90713 (19), and GSE143383 (20). The GSE12368 dataset is based on the GPL570 platform and contains 12 ACC samples (5 male samples) and 6 normal samples (0 male samples). GSE90713 dataset is based on the GPL15207 platform and contains 58 ACC samples and 5 normal samples. GSE143383 dataset is based on the GPL16043 platform and contains 57 ACC samples (17 male samples) and 5 normal samples (3 male samples).

### Data preprocessing and identification of differentially expressed genes (DEGs)

The DEGs between ACC and normal samples in each GEO dataset were screened using GEO2R (http://www.ncbi.nlm.nih.gov/geo/geo2r). GEO2R is an online web tool that compares the datasets in a GEO series to identify DEGs under different conditions. Genes with more than one probe set were averaged and probe sets without corresponding gene symbols were removed. |log_2_FC (fold change) |≥1 and adjusted P-values (adj. P) <0.05 were considered statistically significant.

### GO and KEGG enrichment analyses of DEGs

GO is a main bioinformatics tool to analyze the biological process (BP), cellular component (CC) and molecular function (MF) of genes (21, 22). KEGG is an online database for gene functional enrichment (23). GO and KEGG enrichment analysis of DEGs were performed using DAVID (version 2021) (24) database (https://david.ncifcrf.gov/). P<0.05 was considered statistically significant.

### PPI network construction and module analysis

Search Tool for the Retrieval of Interacting Genes (STRING) (version 11.5) (25) online database (http://www.string-db.org/) was used to construct the protein-protein interaction (PPI) network, thereby providing insights into the occurrence and development of diseases. Then, the PPI network was visualized by the Cytoscape (version 3.9.1) software (26) (http://www.cytoscape.org/) to further analyze the complex relationships among the DEGs. Significant modules in the PPI network were identified using plug-in Molecular Complex Detection (MCODE) (version 2.0.0) (27) (Parameters were the default values) of Cytoscape. Subsequently, the GO and KEGG analyses were performed for genes in the most significant module using DAVID.

### Hub genes selection and analysis

The plug-in cytoHubba (28) of Cytoscape was used to screen hub genes based on the PPI network. Cytohubba ranks the importance of each gene according to different algorithms. Among 12 computing methods, we selected the maximal clique centrality (MCC) method to identify hub genes. Then, the BP analysis of hub genes was visualized using plug-in Biological Networks Gene Oncology tool (BiNGO)(version3.0.3) (29) of Cytoscape. To verify the roles of hub genes, the FPKM gene expression profiles of GDC TCGA Adrenocortical Cancer (ACC) (14 datasets) and CTEX (11 datasets) were downloaded using the University of California, Santa Cruz (UCSC) Xena browser (30) (http://genome-cancer.ucsc.edu). The datasets contained 79 ACC samples and 128 normal samples. The violin plot was used to show the gene expression of hub genes in ACC and normal adrenal samples. The overall survival analyses and disease free survival analyses of hub genes were performed using Kaplan-Meier curve in cBioPortal (version 5.2.2) (31, 32)(http://www.cbioportal.org/) online platform. The relationship between hub genes and tumor stage in ACC patients was presented using stage plot in Gene Expression Profiling Interactive Analysis (GEPIA) (33) (www.gepia.cancer-pku.cn).

## Results

### Identification of DEGs

**Figure 1:**
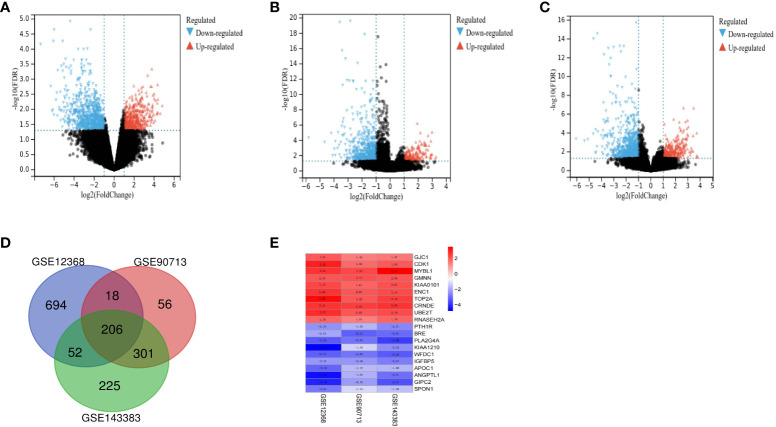
Identification of DEGs between ACC and normal tissues. The volcano plots of DEGs for dataset GSE12368 **(A)**, GSE90713 **(B)** and GSE143383 **(C)**. **(D)** The Venn diagram of the overlapping DEGs among the three datasets. **(E)** The expression heatmap of the top 10 up-regulated and top 10 down-regulated DEGs.

A total of 127 ACC and 16 normal tissues were obtained from the three GEO datasets. Using |log_2_FC|≥1 and adj. P <0.05 as cut-off criteria, DEGs (970 in GSE12368, 581 in GSE90713 and 784 in GSE143383) were identified, including 397 up-regulated and 573 down-regulated genes in GSE12368 dataset, 161 up-regulated and 420 down-regulated genes in GSE90713 dataset, 250 up-regulated and 534 down-regulated genes in GSE143383 dataset. The distributions of DEGs in three datasets are shown in the volcano plots (**Figures 1A–C**), respectively. The overlap among the three datasets contained 206 genes as shown in the Venn diagram (**Figure 1D**), including 72 up-regulated and 134 down-regulated DEGs (**Table 1**) between ACC tissues and normal tissues. The expression heatmap of the top 10 up-regulated DEGs and top 10 down-regulated DEGs is shown in **Figure 1E**.

**Table 1:** Two hundred and six differentially expressed genes (DEGs) were identified and confirmed from three profile datasets, including 72 up-regulated genes and 134 down-regulated genes in the ACC tissues, compared to normal tissues.

Regulation	DEGs (gene symbol)
Up-regulated	TPX2, DEPDC1B, CCNB1, GINS1, ANLN, BIRC5, PLEKHG4, CDK1, KIF11, RACGAP1, CDCA4, C12orf76, TMEM161B, CDC6, KIF2A, SMC4, PLXNC1, AURKA, DHFR, MAD2L1, TMPO, STC1, GAS2L3, SULF2, C5orf34, TYMS, TMEM106C, ZWINT, NDC80, H2AFZ, RFC4, CCNA2, TMEM132A, BUB1, PBK, SPATS2, TRIP13, CDCA5, UBE2T, ATF7IP, CRNDE, ANGPT2, MYBL1, TIGD7, DIAPH3, GGH, SHCBP1, ASPM, GADD45A, MDM2, UBE2C, MND1, CCNB2, PRC1, CEP55, TOP2A, FANCI, ZNF367, RAD51AP1, RNASEH2A, DTL, HMMR, GJC1, GMNN, ENC1, CENPK, ZNF404, IGFBP3, KIAA0101, CDKN3, NCAPG, ESM1
Down- regulated	ANKS1A, MMP2, MUM1L1, IGF1, GOLM1, PHYHIP, NFIA, NR2F1, KIAA1210, SLCO2B1, TEK, CD55, SERPINB9, MRPL33, SLCO2A1, SERPING1, PARVA, SLC40A1, CRHBP, ALAD, ZNF331, EBPL, IGSF11, ZBED1, SLITRK4, CYP11B2, ITGA8, TLE2, STAT5A, DUSP26, SORBS2, USP13, STEAP4, KIAA1217, OMD, FBLN1, SLC16A9, PDGFD, SPON1, PLA2G4A, LMOD1, ABCC3, C1GALT1C1, ACADVL, TBXAS1, HOXA5, GALM, CTH, NPY1R, PLCXD3, ECHDC3, LUZP2, KLHL4, USP53, LIMS1, DNAJC12, GRAMD4, C7, IGFBP5, CTGF, EPHX2, CREM, KCNQ1, NR2F2, ANGPTL1, CREBL2, PLIN1, TAGLN, EMCN, KCNK2, FOSL2, TMEM150C, DLG2, AXL, SHE, GIPC2, OGN, ACADSB, MYLK, FZD5, IGFBP6, SLC37A2, FNDC5, ARMC9, HS6ST1, CXCL12, GLUL, EHD3, EFEMP2, ALDH1A1, ADORA3, AEBP1, NEXN, NIT1, WISP1, CBLN4, INMT, BRE, SEC62, TLR4, FMO2, ZNF185, CDC42EP4, RBMS3, CASP9, ADH1B, OLFML3, CAB39L, PDGFRA, CDH19, SIRT1, FAM65C, C11orf96, RSPO3, CPT1A, SREBF1, PRELP, WFDC1, NANOS1, PTH1R, CYBRD1, CRISPLD2, ATP1B3, C2orf40, AOX1, DAPL1, PHF11, HGF, APOC1, C11orf54, FLVCR2, PARM1, PTGIS, FBLN5

### KEGG and GO enrichment analyses of DEGs

**Figure 2:**
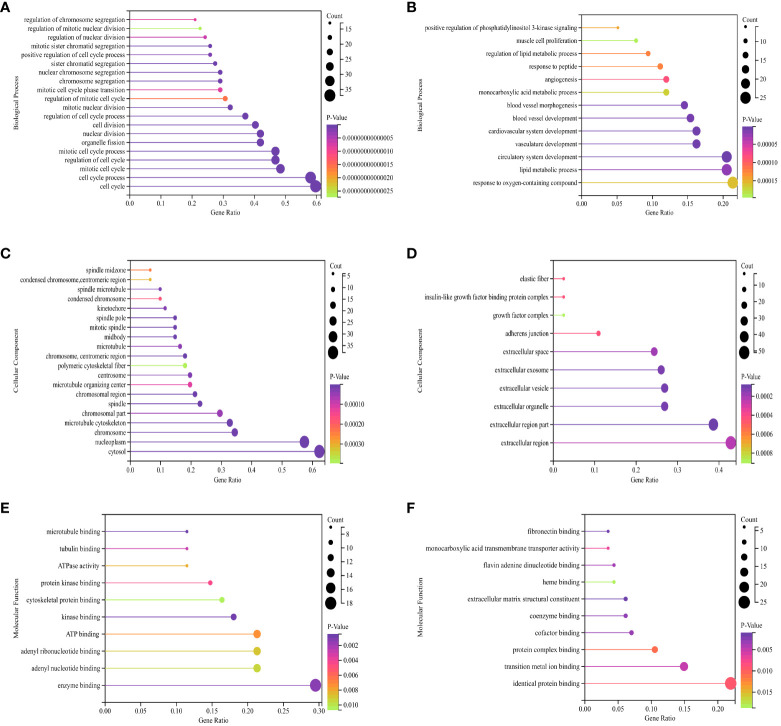
GO (Gene Ontology) pathway enrichment analyses of DEGs (differentially expressed genes). **(A)** Biological process for up-regulated DEGs. **(B)** Biological process for down-regulated DEGs. **(C)** Cellular component for up-regulated DEGs. **(D)** Cellular component for down-regulated DEGs. **(E)** Molecular function for up-regulated DEGs. **(F)** Molecular function for down-regulated DEGs. The X-axis represents the ratio of enriched pathways to total pathways, while the Y-axis displays the names of enriched pathways. “Count” indicates the number of enriched pathways, which corresponds to the size of the dot. The color indicates the P-value, with darker shades indicating smaller P-value.

**Figure 3:**
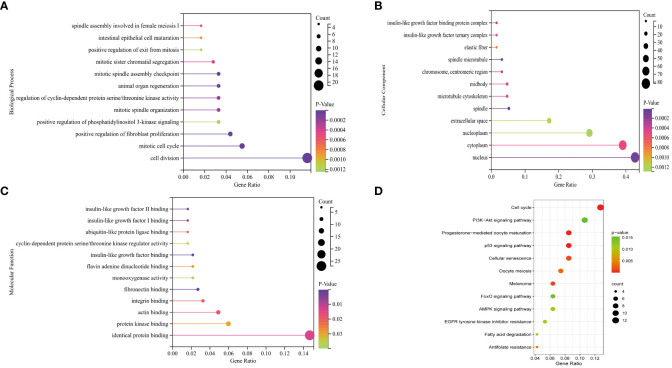
GO (Gene Ontology) pathway enrichment analyses and KEGG (Kyoto Encyclopedia of Genes and Genomes) pathway enrichment analyses of DEGs (differentially expressed genes). **(A)** Biological process for up-regulated and down-regulated DEGs. **(B)** Cellular component for up-regulated and down-regulated DEGs. **(C)** Molecular function for up-regulated and down-regulated DEGs. **(D)** Bubble plot of KEGG for up-regulated and down-regulated DEGs.

**Figure 4:**
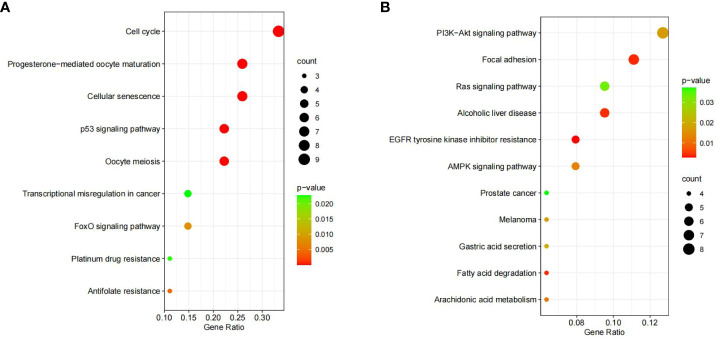
KEGG (Kyoto Encyclopedia of Genes and Genomes) pathway enrichment analyses of DEGs (differentially expressed genes). **(A)** Bubble plot of KEGG for up-regulated DEGs. **(B)** Bubble plot of KEGG for down-regulated DEGs.

The BP of the GO enrichment analysis showed that up-regulated DEGs were significantly enriched in cell cycle, cell cycle process, mitotic cell cycle, regulation of cell cycle and mitotic cell cycle process (**Figure 2A**) and down-regulated DEGs were enriched in response to oxygen-containing compound, lipid metabolic process, circulatory system development, vasculature development and cardiovascular system development (**Figure 2B**). In terms of CC, up-regulated DEGs were enriched in cytosol, nucleoplasm, chromosome, microtubule cytoskeleton and chromosomal part (**Figure 2C**), while the down-regulated DEGs were significantly enriched in extracellular region, extracellular region part, extracellular organelle, extracellular vesicle and extracellular exosome (**Figure 2D**). Up-regulated DEGs were significantly enriched in enzyme binding, adenyl nucleotide binding, adenyl ribonucleotide binding, ATP binding and kinase binding in the MF categories (**Figure 2E**), the most significantly enriched MF terms for down-regulated genes were identical protein binding, transition metal ion binding, protein complex binding, cofactor binding, coenzyme binding and extracellular matrix structural constituent (**Figure 2F**). The BP of the GO enrichment analysis showed that up-regulated and down-regulated DEGs were significantly enriched in spindle assembly involved in female meiosis I, intestinal epithelial cell maturation, positive regulation of exit from mitosis, mitotic sister chromatid segregation, mitotic spindle assembly checkpoint, animal organ regeneration, regulation of cyclin-dependent protein serine/threonine kinase activity, mitotic spindle organization, positive regulation of phosphatidylinositol 3-kinase signaling, positive regulation of fibroblast proliferation, mitotic cell cycle, cell division (**Figure 3A**). In terms of CC, up-regulated and down-regulated DEGs were enriched in insulin-like growth factor binding protein complex, insulin-like growth factor ternary complex, elastic fiber, spindle microtubule, chromosome, centromeric region, midbody, microtubule cytoskeleton, spindle, extracellular space, nucleoplasm, cytoplasm, nucleus (**Figure 3B**). Up-regulated and down-regulated DEGs were significantly enriched in insulin-like growth factor II binding, insulin-like growth factor I binding, ubiquitin-like protein ligase binding, cyclin-dependent protein serine/threonine kinase regulator activity, insulin-like growth factor binding, flavin adenine dinucleotide binding, monooxygenase activity, fibronectin binding, integrin binding, actin binding, protein kinase binding, identical protein binding in the MF categories (**Figure 3C**). According to KEGG pathway enrichment analysis, up-regulated DEGs were mainly enriched in cell cycle, progesterone-mediated oocyte maturation, p53 signaling pathway, cellular senescence, oocyte meiosis (**Figure 4A**), while the down-regulated DEGs were significantly enriched in PI3K-Akt signaling pathway, focal adhesion, Ras signaling pathway, Alcoholic liver disease, EGFR tyrosine kinase inhibitor resistance (**Figure 4B**). Up-regulated and down-regulated DEGs were significantly enriched in cell cycle, PI3K-Akt signaling pathway, progesterone-mediated oocyte maturation, p53 signaling pathway, cellular senescence, oocyte meiosis, melanoma, FoxO signaling pathway, AMPK signaling pathway, EGFR tyrosine kinase inhibitor resistance, fatty acid degradation, antifolate resistance (**Figure 3D**).

### PPI network construction and module analysis

**Figure 5:**
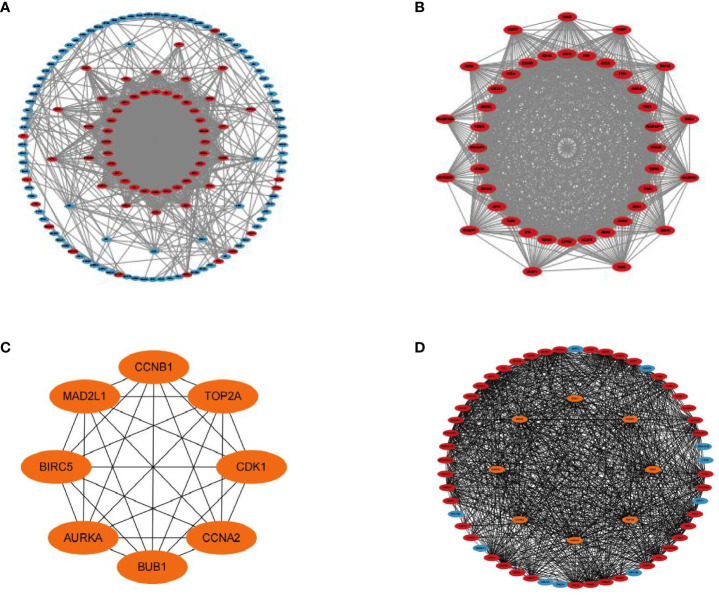
PPI networks of DEGs. **(A)** Up-regulated and down-regulated genes in PPI network (red nodes mean up-regulated genes, and blue nodes mean down-regulated genes). **(B)** The most significant module in PPI network. **(C)** Hub genes in PPI network. **(D)** Hub genes and other genes in PPI network (orange nodes mean hub genes).

The PPI network of the DEGs with 150 nodes and 1094 edges was constructed (**Figure 5A**). In addition, we obtain top three modules using MCODE in Cytoscape, and the genes of three modules were shown in **Table 2**. The most significant module is shown in **Figure 5B** and the PPI network of this module consisted of 41 nodes and 780 edges. GO and KEGG pathway enrichment analysis of DEGs involved in the most significant module were also analyzed using DAVID and results were mainly enriched in cell cycle process, cell cycle, mitotic cell cycle process, nuclear division, progesterone-mediated oocyte maturation (**Table 3**).

**Table 2:** The top three significant modules.

Modules	Nodes	Edges	Genes
Module1	41	780	SMC4, CDCA5, ZWINT, TYMS, KIAA0101, HMMR, PBK, CENPK, DEPDC1B, CDC6, AURKA, MND1, FANCI, TOP2A, CCNA2, GINS1, CDK1, NDC80, CCNB1, CEP55, CCNB2, MAD2L1, TRIP13, BIRC5, CDKN3, UBE2T, ANLN, KIF11, SHCBP1, RFC4, UBE2C, DTL, RNASEH2A, RAD51AP1, TPX2, ASPM, PRC1, NCAPG, BUB1, GMNN, RACGAP1
Module2	6	12	ANGPT2, CTGF, MMP2, HGF, IGFBP3, TEK
Module3	4	5	STAT5A, TLR4, CXCL12, PDGFRA

**Table 3:** GO and KEGG pathway enrichment analysis of DEGs in the most significant module.

pathway ID	Pathway description	Count in gene set	FDR
GO:0022402	Cell cycle process	32	3.72E-27
GO:0007049	Cell cycle	33	1.02E-24
GO:1903047	Mitotic cell cycle process	27	1.02E-24
GO:0000280	Nuclear division	24	3.76E-24
GO:0048285	Organelle fission	24	2.11E-23
GO:0000278	Mitotic cell cycle	27	5.60E-23
GO:0051301	Cell division	23	1.16E-20
GO:0051726	Regulation of cell cycle	27	2.02E-20
GO:0007067	Mitotic nuclear division	19	1.12E-19
GO:0000819	Sister chromatid segregation	17	6.54E-19
Hsa04914	Progesterone-mediated oocyte maturation	7	1.20E-06
Hsa04110	Cell cycle	7	2.13E-06
Hsa04114	Oocyte meiosis	6	5.75E-05

Annotation: FDR (false discovery rate, which corrects the P-value by using multiple hypothesis tests on the P-value to reduce the false positive rate).

### Hub gene selection and analysis

**Figure 6:**
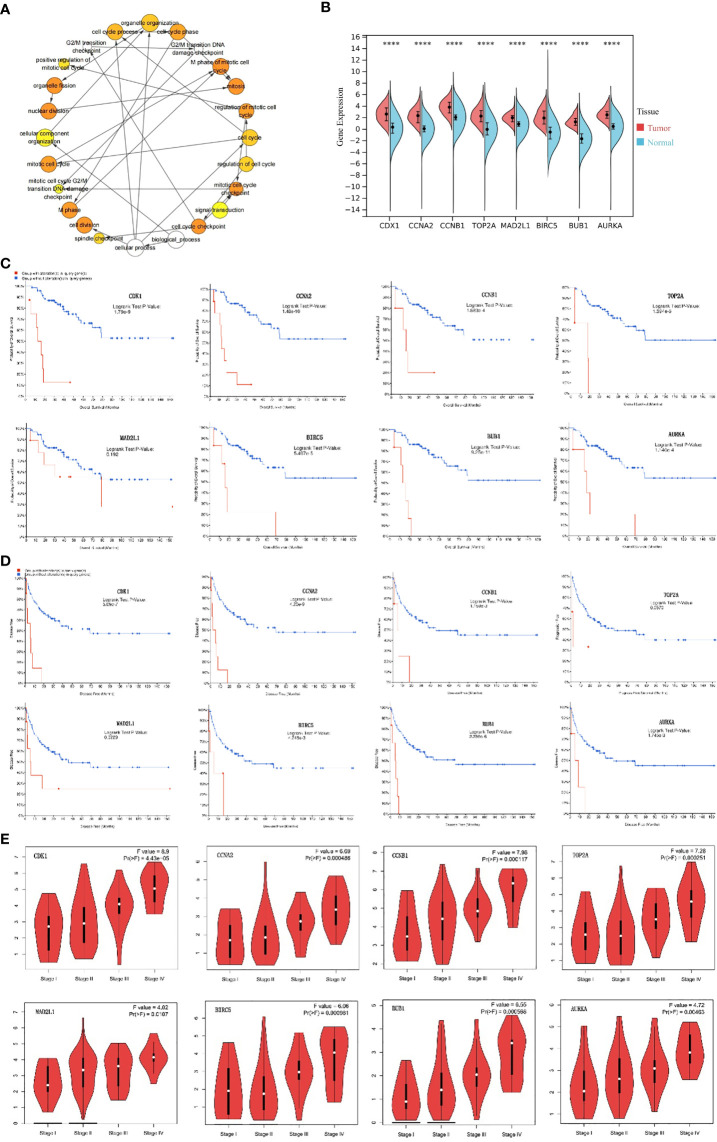
Analysis of hub genes. **(A)** The biological process analysis of hub genes. The color depth of nodes refers to the corrected P-value of ontologies. The size of nodes refers to the numbers of genes that are involved in the ontologies. P<0.0001 was considered statistically significant. **(B)** Violin plot of eight hub genes. ****: P<0.0001. Red represents the expression levels of hub genes in ACC tissues, while blue represents the expression levels of hub genes in normal tissues. **(C)** Overall survival analyses of hub genes. Logrank test P-value <0.05 was considered statistically significant. **(D)** Disease Free survival analyses of 7 hub genes and Progression Free survival analyses of TOP2A. The red line represents the survival rate of a group with alteration in query gene, while the blue line represents the survival rate of a group without alteration in query gene. Logrank test P-value <0.05 was considered statistically significant. **(E)** Correlation between hub genes and tumor stage in ACC patients (GEPIA). ACC, adrenocortical carcinoma.

A total of eight genes were identified as hub genes using the cytoHubba in Cytoscape. They were CDK1, CCNA2, CCNB1, TOP2A, MAD2L1, BIRC5, BUB1 and AURKA. The PPI networks of hub genes and its interaction and other genes were shown in **Figures 5C, D** respectively. All eight hub genes were up-regulated and had high degree of connectivity in the network (**Table 4**). The abbreviations, full names, aliases and functions for the hub genes are shown in **Table 5**. The BP analysis of hub genes was visualized using BiNGO and the result showed that these hub genes were significantly enriched in nuclear division, mitosis, M phase of mitotic cell cycle and cell cycle process (**Figure 6A**). To verify the roles of hub genes, the gene expression profiles of GDC TCGA and CTEX were downloaded. The datasets contained a total of 79 ACC samples and 128 normal samples. The violin plot was used to show the differential gene expression of hub genes in ACC and normal samples (**Figure 6B**). Subsequent survival analysis of these hub genes was performed using cBioPortal, revealing that ACC patients with alterations in CDK1, CCNA2, CCNB1, BIRC5, BUB1, and AURKA (depicted by the red solid line) demonstrated significantly lower overall survival rates and disease free survival rates compared to patients without alterations in these genes (depicted by the blue solid line). Similarly, overall survival was reduced in ACC patients with TOP2A alteration. The results of TOP2A progression free survival demonstrated that ACC patients with TOP2A alterations had lower progression free survival (**Figures 6C, D**). The relationship between the expression of hub genes and ACC tumor stage is showed in **Figure 6E**, where higher ACC tumor stage is associated with higher hub gene expression.

**Table 4:** Hub genes with high degree of connectivity.

Gene symbol	Degree	Type
CDK1	53	Up
CCNA2	50	Up
CCNB1	50	Up
TOP2A	47	Up
MAD2L1	47	Up
BIRC5	47	Up
BUB1	46	Up
AURKA	46	Up

**Table 5:** Functional roles of 8 hub genes.

No.	Gene symbol	Full name	Aliases	Function	References
1	CDK1	Cyclin dependent kinase 1	CDC2; CDC28A; P34CDC2	As a key regulator of the cell cycle, CDK1 is a potent therapeutic target for inhibitors in cancer treatment	(34–37)
2	CCNA2	Cyclin A2	CCN1; CCNA	CCNA2 belongs to the cell cyclin family and is an oncogene of various solid tumors, such as clear cell renal cell carcinoma and breast cancer	(38, 39)
3	CCNB1	Cyclin B1	CCNB	CCNB1 is associated with tumor immune infiltration, and its overexpression is associated with poor prognosis of breast cancer and hepatocellular carcinoma	(40–42)
4	TOP2A	DNA topoisomerase II alpha	TOP2; TP2A; TOPIIA; TOP2alpha	The enzyme encoded by TOP2A functions as the target for several anticancer agents and a variety of mutations in TOP2A have been associated with the development of drug resistance	(43–45)
5	MAD2L1	Mitotic arrest deficient 2 like 1	MAD2; HSMAD2	MAD2L1 has become a potential biomarker for various cancers such as hepatocellular carcinoma and cholangiocarcinoma	(46)
6	BIRC5	Baculoviral IAP repeat containing 5	API4; EPR-1	BIRC5 is highly expressed in most tumors and it regulates the progression of cancer development	(47, 48)
7	BUB1	BUB1 mitotic checkpoint serine/threonine kinase	BUB1A; BUB1L; hBUB1	Mutation in BUB1 is associated with several cancers such as breast cancer and hepatocellular carcinoma	(49–51)
8	AURKA	Aurora kinase A	AIK; ARK1; AURA; BTAK; STK6; STK7; STK15; PPP1R47	AURKA may play a role in tumor development and progression	(52, 53)

## Discussion

In this study, we identified 206 DEGs in three GEO datasets, including 72 up-regulated and 134 down-regulated genes. Functional enrichments analyses showed that these DEGs were mainly enriched in cancer-related pathways and functions. The PPI network was constructed using the STRING database, and the analysis of significant modules was performed. Finally, we filtered out eight hub genes, including CDK1, CCNA2, CCNB1, TOP2A, MAD2L1, BIRC5, BUB1 and AURKA. Survival analysis of these hub genes based on the cBioportal confirmed the reliability of the results. In recent years, several studies about DEGs in ACC have been published. For example, a total of 200 DEGs were identified based on three GEO microarray datasets (GSE12368, GSE10927 and GSE90713) (54). KEGG enrichment analysis showed that up-regulated DEGs were enriched in the cell cycle, cell senescence, progesterone-mediated oocyte maturation, oocyte, p53 signaling pathway and folic acid resistance. Nine hub genes (CCNB1, CDK1, TOP2A, CCNA2, CDKN3, MAD2L1, RACGAP1, BUB1 and CCNB2) were identified in the PPI network. CDK1, CCNA2, CCNB1, TOP2A, MAD2L1 and BUB1 were also identified as hub genes in our study. Another study identified eight hub genes (KIF18A, CDCA8, SKA1, CEP55, BUB1, CDK1, SGOL1, SGOL2) associated with poor prognosis in ACC (51), we found that CDK1 and BUB1 existed in our results. Although 2 GEO microarray datasets (GSE12368 and GSE90713) have been analyzed in previous studies, GSE143383 in this study, which contains 57 ACC samples and 5 normal samples, has not been analyzed previously. ACC samples in GSE143383 account for the majority of the samples, the results of the analysis have strong reliability. In addition, most of the hub genes analyzed in this study are consistent with those obtained in previous studies, which further confirmed the role of these genes in the progression of ACC and also provided theoretical basis for the development of clinical targeted drugs. As a key regulator of the cell cycle, the mRNA expression of CDK1 has been found to be up-regulated in a variety of tumor tissues, including ACC, and high expression of the gene was associated with poor prognosis in patients (34–37). CDK1 interacted with Centromere Protein F (CENPF) to enhance the G2/M phase transition of mitosis and cell proliferation of ACC cells. Recently, Liwen Ren et al. (55) found that CDK1 can be used as an important therapeutic target for ACC by regulating the epithelial-to-mesenchymal transition (EMT), G2/M checkpoint and PANoptosis. Quantitative high-throughput drug screening on ACC cells in a preclinical study has revealed a novel combination of CDK inhibitor and maternal embryonic leucine zipper kinase (MELK) inhibitor, which can effectively target a variety of molecules related to ACC aggressiveness (56). In the future, clinical trials of drugs for ACC patients are expected to improve their survival rate. CCNA2 belongs to the cell cyclin family and is an oncogene of various tumors, such as clear cell renal cell carcinoma (38) and breast cancer (39). CCNB1 promoted tumor cell proliferation and metastasis in hepatocellular carcinoma (HCC) (40) and gastric cancer (41), and was found to be overexpressed in ACC patients with distant metastasis and was significantly associated with mortality (42). The enzyme encoded by TOP2A is the main target for several anticancer agents. TOP2A promotes the malignant progression of a variety of tumors, and its expression in ACC is significantly associated with poor overall survival (43), and is a therapeutic target for ACC (44), as well as for HCC (45). The overexpression of MAD2L1 is associated with HCC stage, adjacent organ invasion and poor prognosis (46) and the gene has become a potential biomarker for various cancers. BIRC5 is a member of apoptosis inhibitor family, its overexpression is related with the occurrence and progression of multiple cancer types and is significantly associated with worse overall survival and increased mortality in a variety of cancers, including ACC (47, 48). BUB1 plays a key role in chromosome arrangement during the spindle assembly checkpoint and mitosis (49) and it promotes the initialization and development of bladder cancer by regulating the STAT3 signaling (50). It has been shown that elevated BUB1 expression associated with the abundance of tumor-infiltrating mast cells in ACC patients is significantly related with poor prognosis (51). AURKA belongs to the serine/threonine kinases family and its activation is required for the cell division process. AURKA is highly expressed in tumor tissues and is a potential target for cancer therapy (52). Its overexpression has been associated with poor prognosis and resistance to chemotherapy in ACC patients. Therefore, targeting AURKA could be a promising therapeutic strategy for ACC. It has been shown that ACC with TP53 somatic variants has atypical mitosis, which may be related to M phase dysregulation mediated by AURKA (57). ACC was reported to be a particularly strong indicator of TP53 germline mutations, with 67% of tested patients showing a mutation (58). The p53 R337H, a common pathogenic variant found in many ACC patients. One study examined the p53 status of 36 Brazilian patients with ACC and found that 35 of 36 patients had p53 germline point mutation that codes for R337H amino acid substitution (59). A recent study found that among 42 DEGs related to ferroptosis in ACC, especially AURKA was significantly associated with poor prognosis of ACC and according to a pan-cancer analysis, AURKA may play an important role in tumor immunity and tumor micro-environment, and it is expected to become a predictive biomarker for a variety of cancers (53). The Wnt/beta-catenin pathway, which is a very important pathway (17), is related to steroidogenesis excess observed in most of ACCs. Targeting AURKA decreases cell proliferation, however, it increases activation of the Wnt/beta-catenin pathway, which in turn, can contribute to ACC progression. *In vitro* studies have shown that inhibition of AURKA and Wnt/beta-catenin pathways can reduce the growth of adrenal cortical cancer cells, so targeting AURKA and beta-catenin may be a strategy to treat ACC (60). Several inhibitors of AURKA have been developed and tested in preclinical and clinical studies. For example, the small molecule inhibitor MLN8054 has shown promising results in inhibiting AURKA activity and suppressing tumor growth in xenograft models (61). Another inhibitor, alisertib, has demonstrated efficacy in inhibiting AURKA, resulting in the induction of cell cycle arrest and apoptosis (62, 63). In addition to AURKA, other potential therapeutic targets for ACC have been identified in the literature. These include IGF2, TP53, and CTNNB1, which are known to be dysregulated in ACC and play important roles in tumor growth and progression (64–66). Targeting these genes or their downstream signaling pathways could provide additional treatment options for ACC patients. The genes and pathways mentioned above may seem promising, however, the truth is that even though this information is available, and we know that targeting cell cycle regulators, for example, might have an effect in ACCs, we also know that most of these drugs are toxic to their effective levels and therefore, are not suitable for patient treatment. And that is why, there is no effective treatment for ACCs yet (specially in advanced stages). Therefore, it is necessary to find highly specific drugs that are effective to treat ACCs with decreased levels of toxicity to the patient. Further research is needed to explore combination therapies targeting multiple dysregulated genes in ACC. By targeting these hub genes, we may improve the outcomes for ACC patients and provide new opportunities for personalized medicine in this rare and aggressive cancer. However, our study had several limitations. First, taking the intersection of DEGs from different datasets was not the best approach. This defect may have led to a bias in the final results. Second, all of the eight hub genes analyzed in this study have been reported to be related with the progression and prognosis of ACC patients, so this study is not innovative enough. Moreover, further studies are needed to confirm the influence of these genes and pathways in ACC.

## Conclusions

In the present study, we not only identified 206 significant DEGs in ACC, but we found eight hub genes. Violin plot, Kaplan-Meier curve and stage plot of these hub genes confirmed the reliability of the results. Therefore, the results in this study provided reliable key genes and pathways for ACC, which will be useful for ACC mechanisms, diagnosis and candidate targeted treatment. However, further studies are needed to investigate the potential impact of these genes on ACC progression, in order to fully validate their role in ACC.

## Data availability statement

Publicly available datasets were analyzed in this study. This data can be found here: The data that support the findings of this study are available in the GEO database (http://www.ncbi.nlm.nih.gov/geo) under reference numbers [GSE12368, GSE90713, GSE143383].

## Ethics statement

Ethical approval was not required for the study involving humans in accordance with the local legislation and institutional requirements. Written informed consent to participate in this study was not required from the participants or the participants’ legal guardians/next of kin in accordance with the national legislation and the institutional requirements. Written informed consent was not obtained from the individual(s) for the publication of any potentially identifiable images or data included in this article because This manuscript is based on data obtained by analyzing public databases.

## Author contributions

MY wrote the manuscript. MY, YW, XR and MH participated in the analysis process. MY, SL and RL edited the figures and tables. GW and XG revised the manuscript. All authors contributed to the article and submitted and approved the submitted section.
